# Prognostic Nutritional Index for Predicting 3-Month Outcomes in Ischemic Stroke Patients Undergoing Thrombolysis

**DOI:** 10.3389/fneur.2020.00599

**Published:** 2020-06-26

**Authors:** Weiwei Xiang, Xiyi Chen, Weiyi Ye, Jia Li, Xu Zhang, Dewei Xie

**Affiliations:** ^1^Department of Neurology, The First Affiliated Hospital of Wenzhou Medical University, Wenzhou, China; ^2^Department of Cardiovascular and Thoracic Surgery, The Second Affiliated Hospital of Wenzhou Medical University, Wenzhou, China

**Keywords:** ischemic stroke, intravenous thrombolysis, malnutrition, prognostic nutritional index, prognosis

## Abstract

**Objective:** Malnutrition has been reported to be related to adverse prognosis in acute ischemic stroke (AIS) patients. Unfortunately, traditional nutritional assessment tools usually increase the workload of neurologists, which makes them unfeasible in the daily clinic work. We aimed to elucidate the association between the prognostic nutritional index (PNI), an easily obtainable baseline nutritional marker, and 3-month outcomes in AIS patients receiving intravenous thrombolysis (IVT).

**Research methods and procedures:** The present study retrospectively included 405 patients. PNI was calculated as 5^*^lymphocyte count (10^9^ /L) + serum albumin concentration (g/L), and the good prognosis was defined as modified Rankin Scale score of 0–3. The relationship between PNI and clinical parameters was evaluated. The multiple logistic regression model was performed to find out independent predictors of the 3-month outcomes.

**Results:** We found that the patients in the low PNI group had a higher frequency of anemia (12.9 vs. 2.3%, *P* < 0.001) and a higher level of the Controlling nutritional status (CONUT) score (*P* < 0.001). The relationship between PNI and nutrition-related factors, such as body mass index (*r* = 0.208, *P* = 0.001), age (*r* = −0.329, *P* < 0.001), total cholesterol (*r* = 0.268, *P* < 0.001) and hemoglobin concentration (*r* = 0.328, *P* < 0.001), was significant. Low PNI value (adjusted odds ratio: 2.250, confidence interval: 1.192–4.249, *p* = 0.012) stayed as an independent predictor for the poor outcome at three months, after adjustment for potential confounders.

**Conclusions:** The PNI was independently associated with 3-month outcomes in AIS patients undergoing IVT. As an easily obtainable nutritional marker, PNI may be a useful nutritional assessment tool in the clinic work.

## Introduction

Malnutrition has been demonstrated to be correlated to poor prognosis in a variety of diseases, such as heart failure, malignant diseases as well as acute ischemic stroke (AIS) (1–5). Also, studies have shown that nutritional status at admission was independently associated with clinical outcomes in stroke patients (5). As early nutritional intervention would improve the life quality of malnourished patients, it is essential to assess nutritional status in patients with AIS (6).

It is not easy to evaluate the nutritional status of the patients. Many nutritional indicators are too subjective because they are either affected by the examiners' experience or obtained verbally from patients and their relatives. Moreover, collecting all the subjective information would inevitably increase the workload of routine clinical practice. As an objective nutritional marker, prognostic nutritional index (PNI) was easy to calculate using serum albumin concentration and lymphocyte count, which were routine detection index in the blood test. Thus, PNI is more feasible in the clinic work.

As far as we know, it is still unclear whether nutritional status assessed by PNI was useful for predicting the short-term clinical outcome in AIS patients. We believed that nutritional status on admission in AIS patients with intravenous thrombolysis (IVT) could better represent the baseline nutritional status. Because they have a short and relatively uniform onset-to admission time when compared to the AIS patients without IVT, the nutritional status of patients was less likely to be influenced by neurological dysfunction during such a short time. Therefore, we aimed to explore the prognostic significance of PNI in AIS patients receiving IVT in the present study.

## Participants and Methods

### Patients

This study involved AIS patients undergoing IVT with recombinant tissue plasminogen activator (rt-PA) in The First Affiliated Hospital of Wenzhou Medical University. Four hundred and fifty-one patients were admitted to our hospital from June 2013 to September 2018. The neurologists diagnosed AIS by clinical symptoms, signs, brain computed tomography or magnetic resonance imaging; The inclusion criteria were: diagnosis of AIS and treatment with intravenous rt-PA. Exclusion criteria were: baseline modified Rankin Scale (mRS) more than 2; incomplete follow-up; incomplete medical records; active inflammatory diseases; known malignancy and hematological diseases/intravenous infusion of blood and blood products within 24 h after IVT; severe hepatic or renal dysfunction. Overall, 405 patients were retrospectively included in the present study.

### Ethics Approval Statement

The patients with a diagnosis of acute ischemic stroke underwent IVT after informed consent. The study was performed with the approval of our institutional review boards.

### Data Collection

Demographic data and information about age, gender, hypertension, diabetes mellitus, coronary artery disease (CAD), smoking, drinking, and atrial fibrillation were collected and analyzed. Thrombolysis-related information (onset-to-treatment time and hemorrhagic transformation) was also collected. National Institutes of Health Stroke Scale (NIHSS) scores on admission were assessed by experienced clinicians. Hemoglobin concentration, total cholesterol, triglyceride, high-density lipoprotein cholesterol (HDL-C), and Low-density lipoprotein cholesterol (LDL-C) were assessed within 24 h after admission. Anemia was defined according to the World Health Organization criteria as a hemoglobin concentration of <120 g/L in men and <110 g/L in women. PNI was calculated as 5^*^lymphocyte count (10^9^ /L) + serum albumin concentration (g/L), while in the Controlling nutritional status (CONUT) scoring system point values were assigned to different ranges of lymphocyte count, serum albumin concentration as well as total cholesterol. Specifically, serum albumin concentration ≥35.0 (g/L), 0 point; 30.0–34.9, 2 points; 25.0–29.9, 4 points; and <25.0, 6 points; lymphocytes count ≥1.60 (10^9^ /L), 0 points; 1.20–1.59, 1 point; 0.80–1.19, 2 points; and <0.8, 3 points; and total cholesterol ≥180.00 (mg/dL), 0 point; 140.00–179.99, 1 point; 100.00–139.99, 2 points; and <100.00, 3 points. A CONUT score of 5–12 was used to define malnutrition (moderate or severe) in this study. All patients had a stroke classification according to a Trial of Org 10172 in Acute Stroke Treatment (TOAST) system, a computed tomography scan on admission as well as 24 h after it and a magnetic resonance imaging scan during hospitalization. We also evaluated the mRS score over the phone after three months, and the good prognosis was defined as mRS score of 0–3.

### Statistical Analysis

We used Mean ± standard deviation (SD), median with interquartile range (IQR) or number (percentage) to describe the variables. Categorical variables were analyzed by the χ^2^ test. We compared the intergroup difference of continuous variables using the Mann-Whitney *U*-test, Analysis of Variance or t-test. Spearman's correlation analysis was performed to determine the relationship between the PNI and clinical parameters. The multivariate logistic regression model with a forward procedure was performed to find out independent predictors of unfavorable outcomes at three months. Variables achieving univariate *p* < 0.10 were included in multivariate logistic regression analysis. The receiver operating characteristic (ROC) curve was carried out to determine the optimal cutoff value of PNI. All analyses were performed using SPSS software version 22.0 (SPSS Inc., Chicago, IL) and statistical significance was set at a *P* value < 0.05.

## Results

**Figure 1:**
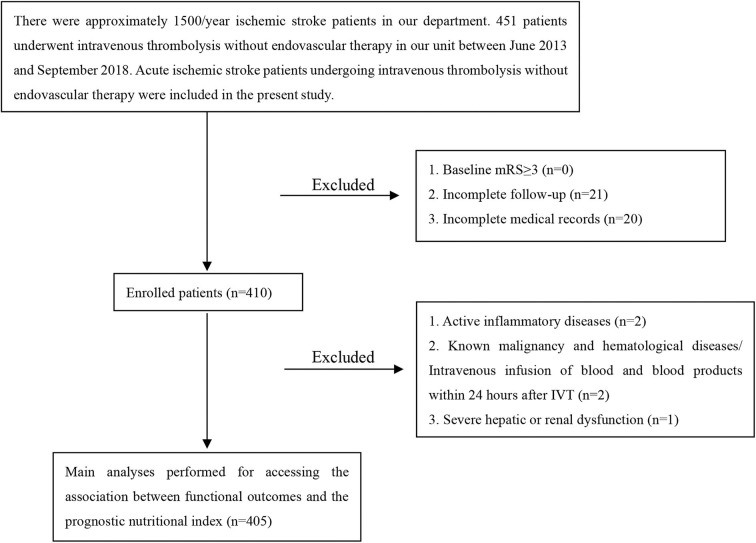
A flow diagram of study participants.

The present study was comprised of 405 AIS patients (Figure 1). The laboratory findings and demographic characteristics were shown in Table 1. The median age of these patients was 66.0 (IQR 16.0) with 48.1 % being male. We established a PNI value higher than 44.15 as the optimal cutoff point for good prognosis (mRS score 0–3). Therefore, the patients were divided into different groups, namely, low PNI and high PNI groups.

**Table 1:** Demographic characteristics and laboratory findings in patients with different PNI (*n* = 405).

**Characteristics**	**Total *n* = 405**	**PNI**		
		**High PNI^#^**	Low PNI^*^	***P***
		***n* = 258**	***n* = 147**	
Age (year), median (IQR)	66 (16)	64 (15)	71 (16)	<0.001
Male, *n* (%)	195 (48.1)	135 (52.3)	60 (40.8)	0.030
Smoking, *n* (%)	152 (37.5)	107 (41.5)	45 (30.6)	0.033
Drinking, *n* (%)	122 (30.1)	83 (32.2)	39 (26.5)	0.261
Hypertension, *n* (%)	294 (72.6)	184 (71.3)	110 (74.8)	0.488
Diabetes mellitus, *n* (%)	112 (27.7)	82 (31.8)	30 (20.4)	0.015
TIA or previous stroke, *n* (%)	42 (10.4)	19 (7.4)	23 (15.6)	0.011
Coronary artery disease, *n* (%)	38 (9.4)	23 (8.9)	15 (10.2)	0.724
Atrial fibrillation, *n* (%)	102 (25.2)	53 (20.5)	49 (33.3)	0.006
NIHSS on admission, median (IQR)	7.0 (8.0)	6.0 (7.0)	8.0 (9.0)	0.001
TOAST classifications, *n* (%)				0.001
Large-artery atherosclerosis	250 (61.7)	161 (62.4)	89 (60.5)	
Small-artery occlusion	36 (8.9)	32 (12.4)	4 (2.7)	
Cardioembolic	108 (26.7)	57 (22.1)	51 (34.7)	
Other and unknown causes	11 (2.7)	8 (3.1)	3 (2.0)	
OTT				0.081
0–180 min	122 (30.1)	71 (27.5)	51 (34.7)	
181–270 min	220 (54.3)	151 (58.5)	69 (46.9)	
271–360 min	63 (15.6)	35 (14.0)	27 (18.4)	
HT, *n* (%)	75 (18.5)	43 (16.7)	32 (21.8)	0.232
mRS score at 3 months, median (IQR)	2.0 (2.0)	1.0 (2.0)	2.0 (3.0)	<0.001
Nutritional indicators				
Anemia, *n* (%)	25 (6.2)	6 (2.3)	19 (12.9)	<0.001
CONUT score	2.0 (2.0)	1.0 (2.0)	3.0 (2.0)	<0.001
^$^Body mass index (kg/m2), mean (SD)	23.64 (3.18)	24.10 (3.02)	22.65 (3.31)	0.001
Laboratory findings				
TC (mmol/L), mean (SD)	4.95 (1.11)	5.16 (1.12)	4.57 (0.99)	<0.001
TG (mmol/L), median (IQR)	1.28 (0.82)	1.43 (0.88)	1.1 (0.68)	<0.001
HDL-C (mmol/L), median (IQR)	1.13 (0.41)	1.13 (0.42)	1.12 (0.42)	0.829
LDL-C (mmol/L), median (IQR)	2.89 (1.07)	3.10 (1.10)	2.61 (0.92)	<0.001
Albumin (g/L), mean (SD)	38.30 (3.60)	39.96 (2.87)	35.39 (2.84)	<0.001
Lymphocyte count (10^9^/L), mean (SD)	1.52 (0.59)	1.74 (0.57)	1.13 (0.38)	<0.001

*TIA, transient ischemic attack; IQR, interquartile range; NIHSS, National Institutes of Health Stroke Scale; TOAST, a Trial of Org 10172 in Acute Stroke Treatment; OTT, onset-to-treatment time; HT, hemorrhagic transformation; TC, total cholesterol; TG, Triglyceride; HDL-C, high-density lipoprotein cholesterol; LDL-C, low-density lipoprotein cholesterol; HC, hemoglobin concentration; PNI, prognostic nutritional index; SD, standard deviation; mRS, modified Rankin Scale; CONUT score, the controlling nutritional status score*;

$ *254 patients had the body mass index*;

# *High PNI value: patients with a PNI value greater than 44.15*;

* *Low PNI value: patients with a PNI value less than or equal to 44.15*.

**Figure 2:**
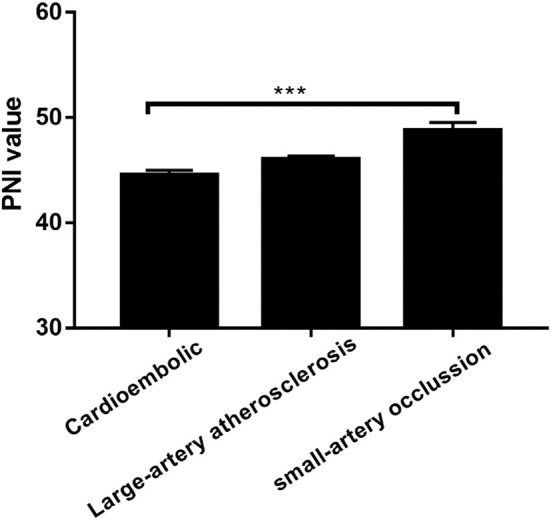
Prognostic nutritional index (PNI) was significantly different between patients with different TOAST classifications. ***P < 0.001; Bar graphs indicate (mean ± SEM) values of PNI.

The low PNI group had a higher value of baseline NIHSS score (*P* = 0.001), 3-month mRS score (*P* < 0.001) and CONUT score (*P* < 0.001) compared to the high PNI group. It was significantly different in respect to the frequency of TIA or Previous stroke (7.4 vs. 15.6%, *p* = 0.011), anemia (2.3% vs. 12.9%, *P* < 0.001) and toast classification (χ^2^ = 15.89, *P* = 0.001) between the two groups. Also, as shown in Table 2, there were correlations between PNI and body mass index (*r* = 0.208, *P* = 0.001), age (*r* = −0.329, *P* < 0.001), total cholesterol (*r* = 0.268, *P* < 0.001), and hemoglobin concentration (*r* = 0.328, *P* < 0.001). Interestingly, PNI value was statistically different among stroke subtypes, with the highest value of PNI in small-artery occlusion stroke and lowest in cardioembolic stroke (Figure 2).

**Table 2:** Relationships between the prognostic nutritional index and clinical parameters.

**Variables**	***r***	***P***
Body mass index (*n* = 254)	0.208	0.001
Age (*n* = 405)	−0.329	<0.001
Total cholesterol (*n* = 405)	0.268	<0.001
Hemoglobin concentration (*n* = 405)	0.328	<0.001

**Figure 3:**
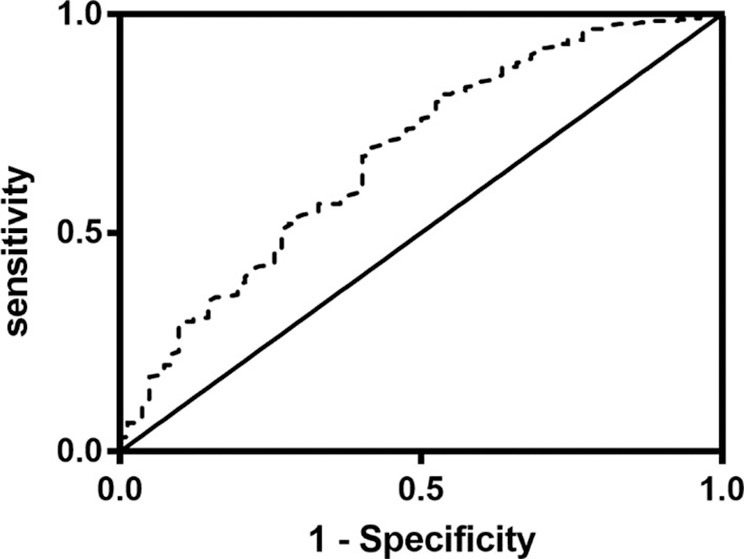
Receiver operating characteristics curve (ROC) of the prognostic nutritional index (PNI) for prediction of 3-month outcomes in acute ischemic stroke patients with intravenous thrombolysis. The value of area under the curve (AUC) for PNI was 0.680 (95% CI:0.613–0.747, *P* < 0.001; cutoff value: 44.15, sensitivity: 70%, specificity: 58.5%).

Univariate regression analyses showed that age, CAD, baseline NIHSS, atrial fibrillation, hemorrhagic transformation, anemia, LDL-C, TOAST classifications, OTT, lymphocyte, albumin, TIA or previous stroke, total cholesterol, high CONUT score and low PNI were significantly associated with the poor outcome at three months (Table 3). As PNI and CONUT score are very similar nutritional assessment tools and use some of the same parameters, we included PNI and CONUT score separately into two multivariate regression models. In model 1, low PNI (adjusted OR 2.250, CI: 1.192-4.249, *p* = 0.0120), age (adjusted OR 1.055, CI:1.024-1.087, *p* = 0.001) and baseline NIHSS score (adjusted OR 1.308, CI:1.227-1.395, *P* < 0.001) were independently associated with the adverse prognosis, while CONUT score of 5-12 (adjusted OR 2.051, CI: 1.081-3.892, *p* = 0.028), age (adjusted OR 1.056, CI:1.025-1.088, *P* < 0.001), and baseline NIHSS score (adjusted OR 1.303, CI: 1.222-1.389, *P* < 0.001) were independent predictors of adverse prognosis in model 2. The ROC curve analysis of PNI for predicting the unfavorable outcome at 3 months yielded an AUC of 0.680 (95% CI:0.613–0.747; *P* < 0.001). The optimal cutoff value of the PNI that best distinguished the favorable/unfavorable outcome at three months was 44.15 (sensitivity: 65.79%, specificity: 75.5%; Figure 3).

**Table 3:** Logistic regression model with predictors of an unfavorable clinical outcome (*n* = 405).

**Variables**	**Unadjusted OR (95% CI)**	***P***	**Model 1**		**Model 2**	
			**Adjusted OR (95% CI)**	***P***	**Adjusted OR (95% CI)**	***P***
Age	1.079 (1.051–1.107)	<0.001	1.055 (1.024–1.087)	0.001	1.056 (1.025–1.088)	<0.001
Male	0.713 (0.436–1.164)	0.176	——	——	——	——
Hypertension	1.438 (0.808–2.557)	0.216	——	——	——	——
Diabetes mellitus	0.949 (0.550–1.638)	0.852	——	——	———	——
TIA or previous stroke	2.169 (1.084–4.340)	0.029	——	——	———	——
Coronary artery disease	2.246 (1.094–4.612)	0.028	——	——	———	——
Smoking	0.778 (0.466–1.298)	0.336	——	——	———	——
Drinking	1.022 (0.603–1.731)	0.936	——	——	———	——
Baseline NIHSS	1.320 (1.241–1.403)	<0.001	1.308 (1.227–1.395)	<0.001	1.303 (1.222–1.389)	<0.001
Atrial fibrillation	4.009 (2.398–6.703)	<0.001	——	——	———	——
HT	3.564 (2.059–6.170)	<0.001	——	——	———	——
TC	0.742 (0.588–0.937)	0.012	——	——	____	——
TG	0.782 (0.567–1.078)	0.134	——	——	———	——
LDL-C	0.779 (0.582–1.043)	0.093	——	——	———	——
HDL-C	0.811 (0.378–1.742)	0.591	——	——	———	——
Albumin	0,921 (0.860–0.986)	0.018	——	——	———	——
Lymphocyte	0.221 (0.129–0.377)	<0.001	——	——	———	——
Anemia	2.852 (1.231–6.608)	0.015	——	——	———	——
CONUT score of 5-12	3.784 (1.677–8.535)	0.001	——	——	2.051 (1.081–3.892)	0.028
TOAST classifications			——	——	——	——
Large-artery atherosclerosis	——	<0.001	——	——	——	——
Small-artery occlusion	0.159 (0.021–1.199)	0.074	——	——	——	——
Cardioembolic	3.550 (2.114–5.963)	<0.001	——	——	——	——
Other and unknown causes	0.558 (0.069–4.485)	0.583	——	——	——	——
OTT		——	——	——	——	——
271–360 min	——	0.050	——	——	——	——
181–270 min	0.499 (0.249–1.004)	0.051	——	——	——	——
0–180 min	0.463 (0.246–0.873)	0.017	——	——	——	——
Low PNI	3.195 (1.938–5.263)	<0.001	2.250 (1.192–4.249)	0.012	——	——

*OR, odds ratio; CI, confidence interval; TIA, transient ischemic attack; NIHSS, National Institutes of Health Stroke Scale; TOAST, a Trial of Org 10172 in Acute Stroke Treatment; OTT, onset-to-treatment time; HT, hemorrhagic transformation; TC, total cholesterol; TG, Triglyceride; HDL-C, high-density lipoprotein cholesterol; LDL-C, low-density lipoprotein cholesterol; PNI, prognostic nutritional index; CONUT score, the controlling nutritional status score. Age, TIA or Previous stroke, Coronary artery disease, Baseline NIHSS, Atrial fibrillation, HT, TC, LDL-C, albumin, lymphocyte, Anemia, TOAST classifications, OTT and low PNI were included in model 1; model 2 included all the variables in model 1 except low PNI was replaced by CONUT score of 5-12. The multivariate regression was performed with a forward procedure. All the variance inflation factor of variables included in the multivariate regression model are less than 10*.

## Discussion

To the best of our knowledge, this is the first investigation to explore the association between PNI and AIS patients undergoing IVT. The main findings were: (1) there was a correlation between the level of PNI and nutrition-related factors, such as age, hemoglobin concentration, total cholesterol and Body mass index; (2) low PNI was independently associated with poor prognosis in the multivariate regression model. (3) the PNI value was significantly different among different stroke subtypes.

PNI was initially reported to be an effective predictor for postoperative complications after surgery (7) and was considered as a predictive nutritional marker for various malignant diseases, such as breast cancer, esophageal cancer, gastric cancer, and so on (8–10). Besides, some studies have demonstrated that as a nutritional marker, PNI was related to the clinical prognosis of patients with cardiovascular diseases, including coronary atherosclerotic heart disease (CAD) and heart failure (11, 12).

It is not a rare condition for AIS patients to suffer from malnutrition: the prevalence of undernutrition was around 33% for acute stroke patients at admission (13, 14). International guidelines recommend nutritional assessment in AIS patients, and several nutritional assessment tools have been reported, such as the Mini-Nutritional Assessment, the Subjective Global Assessment. The subjective parts in these assessments would inevitably increase the workload of routine clinical practice, and it is not easy for neurologists to carry out these nutritional assessments when they are fighting for the precious treatment window for patients. Therefore, as an easily obtainable nutritional marker, PNI is more feasible in the clinic work for patients with ischemic stroke. Importantly, compared with the known predictors of poor outcome for AIS patient, PNI underscores the prognostic significance of nutrition and could motivate the neurologists to pay more attention to the nutritional status of these patients. In the present study.

Studies have shown that there is a high prevalence of anemia in malnourished individuals (15). Consistent with this report, we found that the low PNI group had more patients with anemia, and the correlation between the level of PNI and Hemoglobin concentration was significant. The CONUT score was also an objective nutritional marker comprised of albumin concentration, lymphocyte count, and total cholesterol. Most recently, two small sample size studies have shown that CONUT score on admission was an independent predictor of an unfavorable clinical outcome in AIS patients without IVT (16, 17). To date, the prognostic value of CONUT score has never been verified in the AIS patients with IVT, thereby we also intended to investigate it in our study. As indicated by the results of multivariate regression analysis in Table 3, CONUT score seem to be a good tool for predicting the clinical outcome as well. One of the features of the present study was that CONUT score was lower than reported in AIS patients without IVT. We think the difference may be attributed to the longer onset to admission time of AIS patients without IVT, which is within 7 days of onset. The nutritional status of patients was likely to be influenced by neurological dysfunction, such as disturbance of consciousness, limb weakness, loss of appetite and so on, during such a long time. Interestingly, there was a significant difference in PNI value between different stroke subtypes. It's not clear why small-artery occlusion stroke patients had higher PNI value compared to cardioembolic stroke patients, however, we have also found this association between nutritional status and stroke classifications in previous studies (18–21).

The present study has some limitations. Firstly, as a single-center study with a relatively small sample size, further studies are needed to confirm our results. Secondly, malnutrition is a complex state involving a reduction in protein reserves, weakening of immune defenses and so on. As far as we know, the added value of PNI over lymphocyte and albumin has not been assessed in the previous studies concerning PNI as well as in this study. Further studies will be needed to corroborate the results and evaluate the added value of PNI. Thirdly, in the present study, low PNI showed association with poor prognosis (whether the poor prognosis was defined as mRS of 3-6 or 4-6) in the univariate regression model. However, in the multivariate regression model, PNI showed an association with the dichotomy mRS 0-3 vs. 4-6 but not with mRS 0-2 vs. 3-6. Further studies are needed to testify whether low PNI is more strongly associated with a more severe adverse outcome. Finally, we have only assessed PNI values once, while the changes over time should also be evaluated. However, one previous study has shown that baseline undernutrition independently predicted subsequent undernutrition (22). Thus, we believe that this deficiency would not affect the validity of this study.

## Conclusion

To the best of our knowledge, this is the first study to explore the association between PNI and the three months outcomes in the patients with AIS. Our study suggested that nutritional status assessed by PNI could be an effective prognostic indicator for AIS patients undergoing IVT. As an easily obtainable nutritional marker, PNI may be a useful nutritional assessment tool in the clinic work.

## Data Availability Statement

The datasets presented in this article are not readily available because the authors do not have permission to share the data. Requests to access the datasets should be directed to https://www.wzhospital.cn/wyyy/web/home/default.aspx#.

## Ethics Statement

The studies involving human participants were reviewed and approved by the first affiliated hospital of Wenzhou Medical University. Written informed consent for participation was not required for this study in accordance with the national legislation and the institutional requirements.

## Author Contributions

DX and XZ conceived of the study. DX coordinated the study. WX, XC, and WY collected the data and wrote the manuscript. JL helped with the statistical analyses and commented on the draft. All authors read and approved the final manuscript.

## Conflict of Interest

The authors declare that the research was conducted in the absence of any commercial or financial relationships that could be construed as a potential conflict of interest.
